# Mycobacterium avium Complex Pulmonary Infection in a Patient With an SLC11A1 Mutation: A Rare Case Report and Review of Literature

**DOI:** 10.7759/cureus.50917

**Published:** 2023-12-21

**Authors:** Ali Almontasheri, Adeeb Munshi, Asim Alsaedi, Ali Alsharief, Amr S Albanna

**Affiliations:** 1 College of Medicine, King Saud Bin Abdulaziz University for Health Sciences, Jeddah, SAU; 2 Allergy and Immunology, King Abdullah International Medical Research Center, Jeddah, SAU; 3 Allergy and Immunology, King Abdulaziz Medical City Jeddah, Jeddah, SAU; 4 Infectious Diseases, King Abdullah International Medical Research Center, Jeddah, SAU; 5 Medicine/Infectious Diseases, King Abdulaziz Medical City Jeddah, Jeddah, SAU; 6 Family Medicine, King Abdullah International Medical Research Center, Jeddah, SAU; 7 Family Medicine, King Abdulaziz Medical City Jeddah, Jeddah, SAU; 8 Pulmonary Medicine, King Abdullah International Medical Research Center, Jeddah, SAU; 9 Pulmonary Medicine, King Abdulaziz Medical City Jeddah, Jeddah, SAU

**Keywords:** mutation, slc11a1, infection, mac, mycobacterium

## Abstract

Mycobacterial avium complex (MAC) is one of the non-tuberculous mycobacterium (NTM) that is known to cause pulmonary disease (PD). MAC PD is diagnosed by fulfilling all of the following: presence of respiratory symptoms, imaging studies compatible with pulmonary disease, and isolation of the mycobacterium from either sputum or bronchial wash in symptomatic patients (isolation of at least two sputum specimens or at least one bronchial wash specimen). A mutation in the solute carrier family 11, member 1 (SLC11A1) gene has been associated with Mycobacteria infections, including MAC. Herein, we present a case of a young female diagnosed with pulmonary MAC who was found later to have an SLC11A1 genetic mutation.

## Introduction

Mycobacterial avium complex (MAC) is part of non-tuberculous mycobacterium (NTM), and it is the most common NTM that causes pulmonary disease (PD) [1]. MAC consists of 12 species, with Mycobacterium avium, Mycobacterium intracellulare, and Mycobacterium chimaera being the most common MAC to cause PD [1].

The solute carrier family 11, member 1 (SLC11A1) gene, previously called natural resistance-associated macrophage protein 1 (NRAMP1), has been correlated with immunity because mutation of the gene makes the host more vulnerable to infection with intracellular pathogens such as Salmonella, Leishmania, and Mycobacteria [2].

Herein we present a case of a young female diagnosed with pulmonary MAC who was found later to have an SLC11A1 genetic mutation.

## Case presentation

A 36-year-old female known to have bronchiectasis was diagnosed in a private hospital based on a chest computerized tomography (CT) scan; no investigations were done for her bronchiectasis in the private hospital. She had been diagnosed for more than 10 years with recurrent exacerbation requiring antibiotics and presented to the respiratory clinic for further evaluation of her bronchiectasis. Since the last year, she had a monthly exacerbation in the form of an increase in her baseline cough, sputum production, and change in the color of sputum. She received broad-spectrum antibiotics for every exacerbation with no benefit. 

At the time of presentation, she was complaining of subjective fever, shortness of breath (SOB) worsening from her baseline in the form of SOB with minimal exertion (her baseline, she got SOB with vigorous activity), and cough productive of yellowish sputum of moderate amount also increase from her baseline (her baseline is a dry cough, sometimes productive of minimal amount of yellow/white sputum). She denies any weight loss, loss of appetite, or night sweats. 

Other systematic history was unremarkable. Her family history was remarkable for treated pulmonary tuberculosis (TB) in her six-year-old daughter two years prior to her presentation with nine months of first-line anti-TB medications. She has another daughter (10 years old) with no significant medical history. She had no recent travel history.

Physical examination at the time of presentation showed blood pressure of 121/80 mmHg, heart rate of 85 beats per minute, respiratory rate of 18 breaths per minute, temperature of 37 C, and oxygen saturation of 99% on room air. Chest examination revealed diffuse crackles with no wheeze. A cardiac examination revealed normal heart sounds with no added sounds. The lymph node examination was unremarkable. Another systemic examination was unremarkable. 

Laboratory investigations revealed a white cell count (WBC) of 9.9 x 109/L (4.0-11.0 x 109/L), C-reactive protein (CRP) 14.1 mg/L (0-5 mg/L), and erythrocyte sedimentation rate (ESR) 120 mm/hr (0-20 mm/hr). HIV-1 serology (combination antigen/antibody immunoassay) was requested, which came out negative (Table 1). 

**Table 1:** Laboratory investigations of the patient upon presentation

Variable	Upon presentation	Reference Range
Hemoglobin (g/dl)	12.4	13.0-18.0
White cell count	9.9	4.0-11.0 x 10^9^/L
Neutrophils	6	2-7.5 x 10^9^/L
Lymphocytes	2.1	1.5-4 x 10^9^/L
C-reactive protein (CRP) (mg/L)	14.1	0-5
Erythrocyte sedimentation rate (ESR) (mm/hr)	2.5	0-20
Creatinine (umol/liter)	55	65-112
Bilirubin, total (umol/liter)	9.9	3.4-22.1
Total protein (g/dl)	66	66-83
Albumin (g/dl)	40	39-50
Alanine aminotransferase (IU/liter)	20	7-44
Aspartate aminotransferase (IU/liter)	21	5-34
HIV-1 serology (combination antigen/antibody immunoassay)	Negative	
Immunoglobulin G (IgG) (g/L)	16	6.5-16.2
Immunoglobulin A (IgA) (g/L)	4	0.65-4.21
Immunoglobulin M (IgM) (g/L)	3	0.4-3.45

Acid-fast bacilli (AFB) smear came out positive twice, whereas mycobacterium polymerase chain reaction (PCR) was negative. Sputum culture grew Mycobacterium intracellulare/chimaera, which was identified by using matrix-assisted laser desorption/ionization-time of flight (MALDI-TOF).

The susceptibility testing results were obtained using the microdilution method according to the Clinical and Laboratory Standards Institute (CLSI) guidelines [3]; the organism was sensitive to amikacin and macrolide (azithromycin, clarithromycin) and resistant to linezolid and moxifloxacin (Table 2).

**Table 2:** Antibiotic sensitivity to Mycobacterium intracellulare/chimaera in the sputum culture MIC: minimum inhibitory concentration

Antibiotics	Sensitivity	MIC (ug/ml)
Amikacin	Sensitive	16
Macrolide (azithromycin, clarithromycin)	Sensitive	2
Linezolid	Resistant	32
Moxifloxacin	Resistant	4

**Figure 1:**
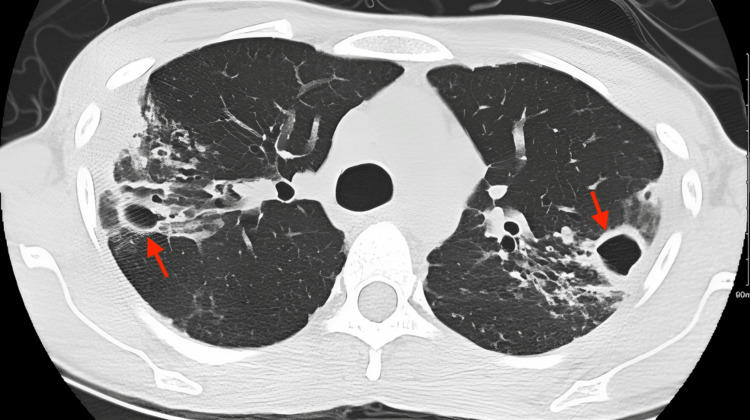
Chest CT showed bilateral nodular bronchiectasis predominantly in the bilateral upper lobes associated with cavitary lesions in the bilateral upper, bilateral lower, and right middle lobes, with the largest in the right upper lobe measuring 2.4 x 1.8 cm. CT: computerized tomography

Chest CT showed bilateral nodular bronchiectasis predominantly in the bilateral upper lobes associated with cavitary lesions in the bilateral upper, bilateral lower, and right middle lobes, with the largest in the right upper lobe measuring 2.4 x 1.8 cm. Diffuse centrilobular nodules and tree-in-bud appearance were more pronounced in the right upper and left lower lobes. Peribronchial wall thickening was noted (Figure 1). 

She was started on ethambutol 800 mg orally once daily, rifampicin 600 mg orally once daily, azithromycin 500 mg orally once daily, and amikacin 10 mg/kg intravenously (IV) three times per week. She received 27 months of ethambutol, rifampicin, and azithromycin (12 months from the first negative sputum culture) and four months of amikacin.

Antibiotics stopped after the patient turned her sputum culture from AFB-positive to AFB-negative, and she improved clinically as her initial symptoms resolved. Also, her radiological findings have been stable during the treatment course, with no further worsening. 

As the response to anti-MAC treatment was slow, the immunology team was consulted to look for primary immunodeficiency as a cause of pulmonary MAC infection. 

More diagnostic tests were requested, including T cell subset analysis, which showed normal B cell count and normal CD4+ and CD8+ T cell counts. Serum immunoglobulin (Ig) levels were also requested, which showed IgG level of 16 g/L (6.5-16.2 g/L), IgA level of 4 g/L (0.65-4.21 g/L), and IgM level of 3 g/L (0.4-3.45 g/L) (Table 1). Genetic testing was offered to the patient, and she accepted and signed the consent. A blood sample withdrawn for whole exome sequencing showed that she had a missense of uncertain significance mutation p.(pro445leu) in the SLC11A1 gene (Table 3).

**Table 3:** Whole exome sequencing of the patient SLC11A1: Solute carrier family 11, member 1

Gene	Variant coordinates	Amino acid change	Zygosity	Type and Classification	Related disorders and mode of inheritance
SLC11A1	NM_000578.3:c.1334>T	P.(pro445Leu)	Heterozygous	Missense uncertain significance (class 3)	Susceptibility to infection by Mycobacterium tuberculosis

Five months after stopping antibiotics, she was seen in the infectious disease clinic. She started to complain of cough in the last three weeks before her presentation, productive yellow sputum, subjective fever, and weight loss of 5 kg. Sputum for AFB was requested and was positive on two occasions. Also, a bronchoscopy with bronchoalveolar lavage (BAL) was done and was positive for AFB. Culture grew again Mycobacterium intracellulare/chimaera from both sputum and BAL, which was identified by using MALDI-TOF. The susceptibility pattern was similar to the previous culture. 

**Figure 2:**
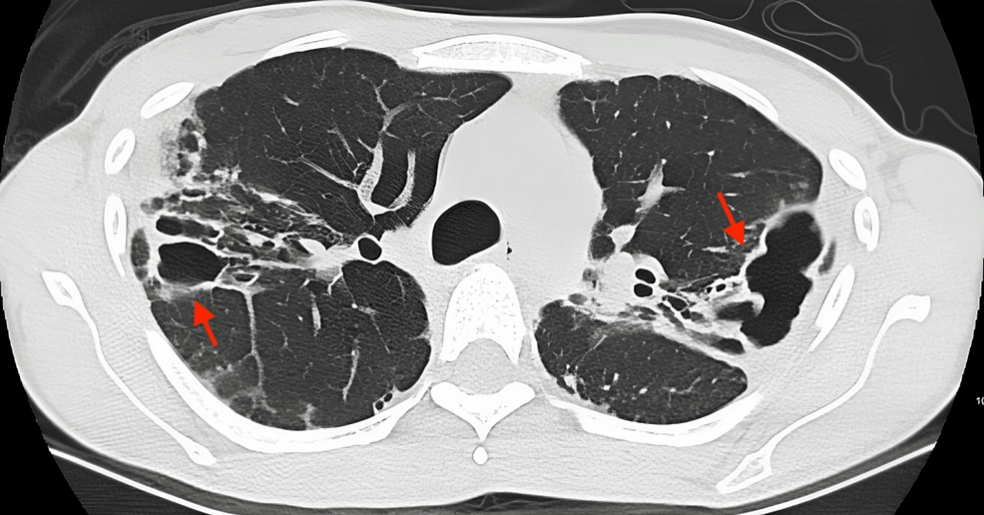
CT chest after the relapse of the infection showing bilateral upper lobes and right middle lobe varicoid and cystic bronchiectasis, demonstrating interval increase in soft tissue thickenings. These findings were consistent with disease progression. CT: computerized tomography

Repeated CT chest showed bilateral upper lobes and right middle lobe varicoid and cystic bronchiectasis, demonstrating interval increase in soft tissue thickenings. These findings were consistent with disease progression (Figure 2). 

She was re-started on ethambutol 800 mg orally once daily, rifampicin 600 mg orally once daily, and azithromycin 500 mg orally once daily. Amikacin inhalation 500 mg twice daily was started, in addition to IV amikacin 10 mg/kg intravenously (IV) three times per week. She received two months of amikacin, then stopped due to early sensorineural hearing loss (SNHL).

The current regimen is ongoing, and the patient is being followed clinically, radiologically, and microbiologically for further improvements. 

## Discussion

A population-based estimate conducted in the United States from 2007 to 2012 revealed that MAC was the most commonly isolated organism (83.6%), and 92.8% of pulmonary isolated were MAC [2]. There are two major clinical phenotypes of MAC-PD: fibrocavitary (FC) and nodular bronchiectatic (NB). The FC phenotype is associated with a worse prognosis than the NB phenotype. However, the NB phenotype is associated with relapse and re-infection [4]. Our patient had FC disease and responded very slowly to anti-MAC treatment. Unfortunately, she had a relapse of her infection five months after completing 12 months of anti-MAC treatment after sputum culture became negative. 

MAC infections can be acquired from the environment. Municipal water sources have been linked as an essential source of MAC-PD [5]. Diagnostic criteria for NTM-PD include MAC requires fulfilling all of the following: the presence of respiratory symptoms, imaging studies compatible with pulmonary disease, and isolation of the mycobacterium from either sputum or bronchial wash in symptomatic patients (isolation of at least two sputum specimens or at least one bronchial wash specimen) [6]. Our patient fulfilled the clinical, radiological, and microbiological criteria with positive sputum culture on two occasions. 

A combination of macrolides, ethambutol, and rifamycin, along with or without injectable aminoglycosides, is recommended for MAC-PD patients after at least 12 months of culture conversion [6,7]. Our patient's prolonged response to treatment with persistent positive sputum culture makes it very reasonable to look for immunodeficiency as a cause of her primary infection. The immunology team was involved, and genetic testing was requested. Whole exome sequencing revealed the presence of SLC11A1 gene mutation.

The mechanism behind the contribution of SLC11A1 to infection could be the deprivation of vacuole pathogens of essential components such as iron, manganese, and cobalt by transferring these important metals out of the phagosomes. Other important roles of SLS11A1 in defending against infections are modulation of phagosome maturation, generation of iron hemostasis, stimulation of innate lymphocytes, and production of proinflammatory cytokines, nitric oxide, reactive oxygen species, and lipocalin 2. When SLC11A1 loses its function, it can lead to a reduction in inflammatory response to infection and disturbance in iron recycling by macrophages [8,9]. 

Tanaka et al. conducted a case-control study among the Japanese population to assess the risk factors for MAC infections. The authors concluded that polymorphisms of the SLC11A1 gene are significantly correlated with pulmonary MAC infections [10]. Another case-control Japanese study by Sapkota et al. found some evidence for an association between NTM disease and the SLC11A1 gene [11]. In a Korean population, Koh et al. found a strong association between NTM-PD, including 18 patients with MAC infection, and polymorphisms of the SLC11A1 gene [12].

## Conclusions

In conclusion, the rare case presented in this study demonstrated the occurrence of pulmonary MAC in a patient with SLC11A1 genetic mutation. The patient had relapsed her infection despite receiving guidelines directing medical therapy toward MAC and stopping treatment after fulfilling the criteria for recovery. Our case demonstrated the importance of undergoing investigations for underlying immunodeficiency in previously labeled immunocompetent patients presenting with pulmonary MAC infection.
